# An RNA-seq atlas of mouse brain areas during fasting and diet-induced obesity

**DOI:** 10.1038/s41597-023-02888-4

**Published:** 2024-01-06

**Authors:** Petar V. Todorov, Anders Bue Klein, Kristoffer L. Egerod, Christoffer Clemmensen, Tune H. Pers

**Affiliations:** 1 Novo Nordisk Foundation Center for Basic Metabolic Research, Faculty of Health and Medical Sciences, University of Copenhagen, Copenhagen, Denmark; 2 Novo Nordisk Foundation Center for Genomic Mechanisms of Disease, Broad Institute of MIT and Harvard, Cambridge, MA 02142 USA

**Keywords:** Hypothalamus, Obesity, Obesity, Gene expression

## Abstract

Mammalian energy homeostasis is primarilly regulated by the hypothalamus and hindbrain, with the hippocampus, midbrain nuclei, and other regions implicated by evidence from human genetics studies. To understand how these non-canonical brain regions respond to imbalances in energy homeostasis, we performed two experiments examining the effects of different diets in male C57BL6 mice. In our first study, groups of six pair-housed mice were given access to chow, high-fat diet or fasted for 16 hours. In our subsequent study, two groups of 10 mice were single-housed and given access to chow or fasted for 24 h. We recorded food intake for each cage, the change in body weight for each animal, and collected hypothalamus, hippocampus, superior colliculus, inferior colliculus, frontal cortex, and zona incerta-centric samples. We performed bulk RNA sequencing on 185 samples and validated them by a series of quality control assessments including alignment quality and gene expression profiling. We believe these studies capture the transcriptomic effects of acute fasting and high-fat diet in the rodent brain and provide a valuable reference.

## Background & Summary

Despite recent successes in treatment options for individuals living with obesity^1–3^, the condition continues to burden millions of individuals worldwide^4,5^. Owing to their preferential access to circulating humoral signals and interoceptive signals from input especially the gut, especially the hypothalamus and dorsal vagal complex in the lower brainstem of the hindbrain have been shown to play a key role in body weight regulation^6^. Cell populations regulating hypothalamic melanocortin 4-receptor signalling have received major attention due to the discovery of rare loss-of-function, obesity-promoting genetic variants in several of the genes coding for receptors and ligands regulating that circuit^6^. However, despite the importance of that well-studied circuit, human genome-wide association studies (GWAS) for obesity have implicated thousands of associated genetic variants, many of which are likely to encode proteins acting through other brain circuits than hypothalamic melanocortin 4-receptor signaling^7–9^.

To better understand which other brain areas besides the hypothalamus regulate body weight, we and others have previously developed gene expression-based computational approaches that have allowed us to agnostically identify other brain areas likely important in obesity pathogenesis^10,11^. In early approaches we used bulk RNA-sequencing (RNA-seq) to successfully show that not only the hypothalamus but several areas other brain areas likely are important too^9^. In more recent approaches, we leveraged hypothalamic and dorsal vagal complex single-cell RNA-sequencing (scRNA-seq) and transgenic mouse models to implicate and validate specific cell populations in these two areas in weight regulation^12–14^. Moreover, in Timshel *et al*^15^., we applied one of our tools, CELL-type Expression-specific integration for Complex Traits (CELLECT), on a total of 727 peripheral and nervous system cell populations from across 17 mouse organs^16^. Interestingly we observed the strongest enrichment in hippocampal cells, followed by significant enrichments in cell populations from the midbrain areas including the superior and inferior colliculus), the hypothalamus and the subthalamic nucleus/zona incerta region.

**Fig. 1:**
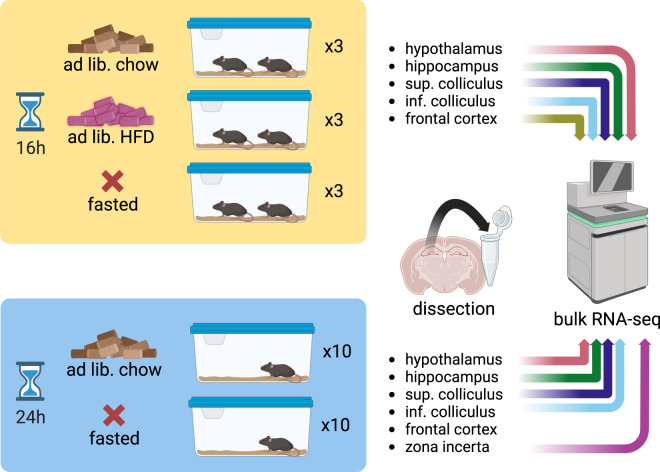
Experimental design of both dietary perturbation studies. Study 1 (yellow), exposed three groups consisting of three cages with two mice each to ad libitum chow, ad libitum high fat diet, or fasting for 16 after which animals were euthanized and samples dissected for bulk RNA-seq. Study 2 (blue), exposed two groups of 10 cages with single-housed mice each to ad libitum chow or fasting for 24 h before collecting samples for bulk RNA-seq. Abbreviations: Ad lib., ad libitum; h, hour; Inf., inferior; sup., superior.

**Fig. 2:**
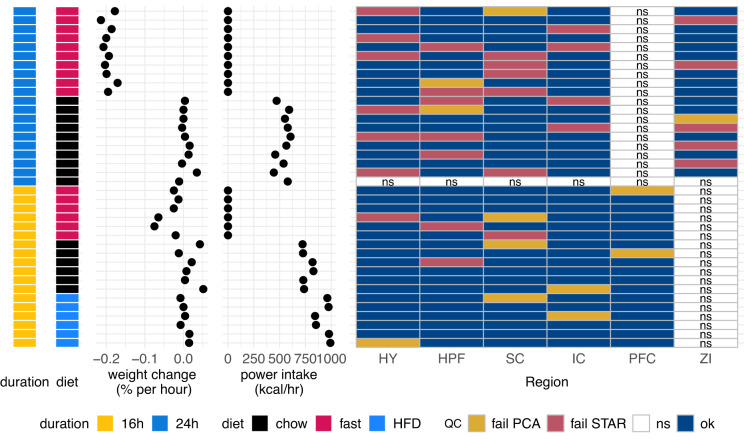
Summary of animal experiments and sequencing results. Each row represents a single animal, colour coded by study duration (yellow for 16 h and blue for 24 h). The next colour coded column represents the diet the animal was exposed to (black for chow, magenta for fasted, and blue for high-fat diet (HFD). The following dot plots show the change in body weight for each animal expressed in units of percent initial weight over duration of hours, and the energy intake per hour of each animal. The grid on the right represents the samples for each animal and their quality control (QC) status. Samples which were not sequenced (ns) are white. Additional abbreviations: h/hr, hour; HY, hypothalamus; HPF, hippocampus; SC, superior colliculus; IC, inferior colliculus; kcal, kilocalories; PFC, prefrontal cortex; QC, quality control; ZI, zona incerta.

The aim of this study was to assess whether dietary perturbations lead to transcriptional changes in gene expression programs within anatomical regions for which we had observed significant overlap between genes in obesity GWAS study loci and the brain scRNA-seq data. Specifically, we aimed to quantify the extent to which fasting and exposure to a high-fat diet (HFD) would exert transcriptional changes in the hypothalamus, hippocampus, superior colliculus, inferior colliculus, and zona incerta of mice. Additionally, as a negative control, we chose to profile the frontal cortex due to the lack of genetic evidence for its role in the phenotype of obesity. We set up two dietary perturbation studies where we exposed mice to the afore-mentioned energetic extremes (Fig. 1), recorded changes in body weight and subsequently isolated the bespoke six brain areas for RNA-seq (Fig. 2).

## Methods

### Animals

C57BL/6 J male mice (8 weeks old) were obtained from (Janvier, FR). All experiments were done at 22 °C (35–55% humidity) with a 12:12‐h light–dark cycle. Mice had free access to water. All experiments were carried out in accordance with regulations regarding the care and use of experimental animals, and the experimental procedures were approved by the Danish Animal Experimentation Inspectorate (2018-15-0201-01457).

#### Fasting and HFD-feeding

At the start of the 16 h study, each of the 18 animals was weighed. Animals were pair-housed in 9 cages and the weight of food in each hopper was also recorded. Three cages were randomized to each experimental condition: kept on a chow diet (Altromin 1324, Brogaarden, DK), fasted, or switched to HFD (D12331; Research Diets). At the end of the study, the weight of food in each hopper and the body weight of each animal was recorded.

Prior to the start of the 24 h study, 20 mice were placed in individual cages supplied with ad libitum chow diet and water and allowed to acclimate to single housing for three days before the start of the experiment. After three days, the mice were randomly and evenly assigned to either remain on chow diet or undergo fasting. The weight of food in the hopper was recorded at the start and end of the 24 h experiment. Mouse body weight was recorded at the start of the acclimation period, at experiment start, and at the end directly before euthanasia and tissue collection.

For the 16 h fasting study the animals were either fasted or exposed to HFD or chow – from 4.30 pm to 8.30 pm (16 hrs), with a 12 hr dark cycle starting at 6 pm. For the 24 h fasting study the animals were either fasted or continued on chow diet from 8.30 am and for 24 hrs, with the dark cycle starting at 6 pm.

### Brain tissue dissection

Following the indicated fasting or HFD feeding periods, mice were euthanized by cervical dislocation. In the 16 h study, we dissected and collected the hypothalamus, hippocampus, superior colliculus, inferior colliculus, and frontal cortex. In the 24 h experiment we collected the same brain regions and the zona incerta. We used anatomical landmarks and coordinates from the Paxinos mouse atlas to determine lateral and ventral-posterior location of the brain regions, which were carefully dissected using microtools. Hypothalamus was crudely dissected as one region, including all nuclei and subregions (i.e. ine scissors and forceps were used to carefully separate the whole hypothalamus from the rest of the brain without compromising its integrity). A left and right sample were dissected and collected from each hemisphere for all regions except the hypothalamus which was taken in one piece. All samples were quickly dissected, frozen in liquid nitrogen, and stored at −80C until further processing. Extensive practice and validation were performed during pilot studies prior to the described experiments.

#### RNA extraction, library preparation and sequencing

For the 16 h study, one sample from the five brain regions of each of the 18 animals was selected for sequencing with the hemisphere chosen at random, for a total of 90 samples. RNA was extracted using RNeasy Micro Kit (Qiagen). Lysis buffer was added to the tissue and dissociated using Stainless Steel Beads (Qiagen) and a TissueLyser II (Qiagen) for 30 sec. This was followed by RNA library construction using the NEBNext® Ultra™ Directional RNA Library Prep Kit for Illumina® including the NEBNext® rRNA Depletion Kit and NEBNext® Multiplex Oligos for Illumina®. The procedures for the kits were followed with no exceptions, and the libraries were quantified using Qubit and qualified using TapeStation measurements. All samples from the 16 h study were sequenced to 51 base pairs (bp) with paired reads on an S2 flowcell in an Illumina Novaseq 6000 instrument.

For the 24 h study we decided to only perform transcriptional profiling of left hemisphere samples and profile the zona incerta instead of the frontal cortex. The choice was made due to the limited differential gene expression in the frontal cortex (PFC) and because differences between hemispheres were not considered the focus of the study. In total, 100 samples representing 5 brain regions collected from 20 animals in the 24 h study brain region and animal were selected for further profiling. All samples from animal 1421 were lost, and in practice 95 samples were randomized in groups of 24 for RNA extraction and library preparation protocol. The first block of 24 samples showed low initial RNA concentration determined by Qubit measurements and TapeStation. It was decided to sequence this block separately on an SP flowcell with paired reads to 61 bp. Remaining blocks of samples were sequenced together on an S2 flowcell to 61 bp paired reads in an Illumina Novaseq 6000 instrument.

### Base calling and alignment

Base calling was performed using Illumina’s bcl2fastq (v2.20.0) software. Demultiplexed reads were assessed with FastQC (v0.12.1). Sequenced reads were then mapped to the GRCm39 build of the *Mus musculus* genome using STAR (v2.7.3a) with an overhang length of 50. The fastq files were provided as input to STAR, using the UNIX “zcat” command to read the compressed files. Quantification mode was set to GeneCounts in the STAR alignment to obtain count data for each gene.

#### Metadata and counts matrix construction

R (v4.2.2) and the tidyverse^17^ (v2.0.0) were used to read Microsoft Excel .xlsx files generated in the lab to produce a table of metadata for each sample. The metadata table contains the following columns:

- “filename” - the stem of both fastq files which were used to generate counts for this sample,
- “name” - the name used for each sample in the counts matrix and downstream analyses “seq_run” - the internal sequencing run ID at our facility
- “seq_sample” - the order of the sample within the sequencing run, “rna_conc_qubit” - the concentration of the extracted RNA from each sample (ng/uL)
- “box_id” - the internal ID of the cage within the animal facility
- “animal_id” - the internal ID of the animal assigned at the animal facility.
- “diet” - the diet the animal was on. Either chow, fast, or HFD.
- “diet_kcal” - the caloric content (kcal/g) of the diet
- “region” - the brain region in this sample. Either hypothalmus (HY), hippocampus (HPF), superior colliculus (SC), inferior colliculus (IC), frontal cortex (PFC), or zona incerta (ZI)
- “hemisphere” - either left, right, or both.
- “experiment_start” - the datetime at start of the experiment
- “time_of_euthanasia” - the datetime at the end of the experiment
- “experiment_duration” - the duration of the experiment (hours)
- “bw_i_pre” - the bodyweight of each animal at the start of the acclimation period (g)
- “bw_i” - the body weight of each animal at the start of the experiment (g)
- “bw_f” - the body weight of each animal before euthanasia (g)
- “bw_change” - the change in body weight between the start and end of the experiment (g)
- “bw_change_pct” - the percentage change in body weight between the start and end of the experiment
- “bw_change_pct_ph” - the rate of change in body weight change (percent per hour)
- “fw_i” - the weight of food in the cage hopper at the start of the experiment
- “fw_f” - the weight of food in the cage hopper at the end of the experiment
- “kcal_intake” - the amount of energy consumed (kcal)
- “power_intake” - the amount of energy consumed per hour (kcal/h)
- “notes” - notes made for each animal or sample during the course of experiment

Notably, the metadata for sample 142.3_SCL_chow and sample 145.4_SCL_fast were corrected to account for the fact that they were erroneously swapped during handling and sequencing. The metadata was written to disk as a comma-separated values (csv) file.

The raw counts for each sample were collected from ReadsPerGene.out.tab and assembled into a 57010 by 185 matrix with ENSEMBL mouse genes as rows and samples as columns. The counts matrix was written to disk as a comma-separated file and deposited in the Gene Expression Omnibus (GEO) database^18^ (GSE236077).

#### Pre-processing

The counts matrix was loaded into R with the read.csv function. The metadata table was loaded and its columns were coerced to factors. A DESeq2 object was created using the function DESeqDataSetFromMatrix, where counts corresponded to the count matrix, colData to the metadata, and the design was set to ~Diet. All genes within the object with cumulative counts less than or equal to 10 were discarded and DESeq function was run on the object. The counts were further processed with a variance stabilizing transform (VST) using the vst function included in the DESeq2 package^19^.

#### PCA to inspect sample gene expression and detect outliers

The prcomp function from the base R library was used to perform principal component analysis (PCA) and ggplot2^17^ was used to generate two dimensional scatter plots with principal component 1 (PC1) on the horizontal axis and PC2 on the vertical axis. PCA computed across all samples and used to detect outliers that extended beyond two standard deviations of PC1 and PC2 for each brain region. Additional visualization was performed by computing PCA on each timepoint and brain region combination.

#### Figures

Final graphics and data visualizations in this manuscript were created using R packages ggplot2 and patchwork, Adobe Illustrator, and BioRender.com.

## Data Records

The data from all 185 bulk RNA-seq samples is deposited in GEO^18^ (GSE236077). All scripts, code, and interactive notebooks are available on the associated on our Zenodo repository^20^.

## Technical Validation

### RNA quality control

For the 16 h study, RNA concentration was quantified using the Qubit instrument and these measurements were used to adjust the concentration of samples to matching concentrations accordingly. All samples, including those with no detectable RNA, were advanced through library preparation and sequencing.

Qubit measurements were also taken of the total RNA extracted from samples in the 24 h study. Here, the first block of 24 samples did not have any detectable RNA as measured by the Qubit instrument, while other blocks showed varying concentrations of RNA in their samples, with only occasional samples lacking detectable RNA. In addition to Qubit measurements, random samples in each of the 4 blocks from the 24 h study were also profiled on the TapeStation instrument. Block 1 from the 24 h study containing all samples without detectable RNA via Qubit also had no RNA detected on the TapeStation. Subsequent blocks had samples in which both peaks for 18 S + 28 S rRNA were present. The total lack of RNA presence in one of the sample blocks indicates poor RNA quality and possible degradation which would impact downstream results. For this reason, Block 1 was sequenced separately and when it did not produce any reads downstream, it was flagged as defective and discarded.

### Sequencing quality control

Following read demultiplexing further quality control metrics were generated with FastQC. All quality metrics from sequencing, demultiplexing, FastQC, and alignment with STAR were aggregated with MultiQC^21^ and manually inspected in the resulting report. All sequencing flowcells performed as expected. S2 flowcells produced between 217-241 Gb/lane, with 87.0-95.4% of bases with Q > 30.

**Fig. 3:**
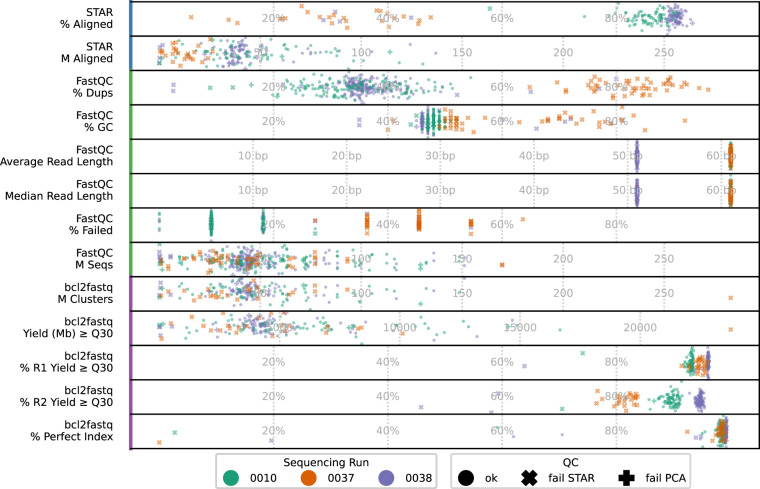
Sequencing quality control results. Each marker represents a sample (in the case of STAR aligned reads) or the forward and reverse reads from that sample (FastQC, bcl2fast). Abbreviations: QC, quality control.

Block 1 from the 24 h study (Fig. 3) with no RNA detected by Qubit or TapeStation featured high rate of fastq read failure (>30%), high GC content (>50%), high rates of duplication (>60%) which further confirmed the lack of successful RNA extraction and library construction.

As expected, the sample block without detected RNA also had a low percentage of aligned reads (<70%) and stood out from all other samples. As a result, we chose to exclude any samples with fewer than 70% of aligned reads (Fig. 2, indicated as “fail STAR”).

### Gene expression profiling

**Fig. 4:**
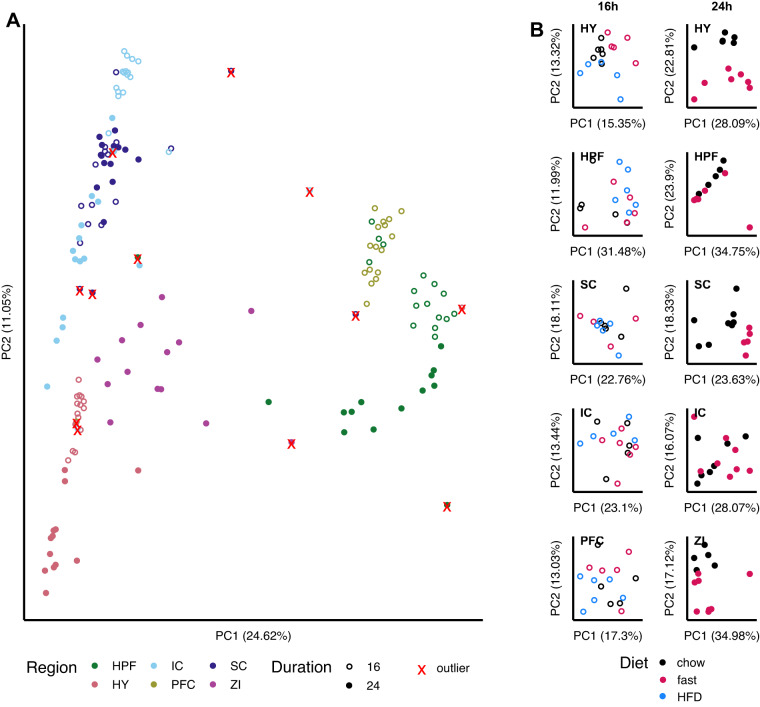
Gene expression profiling and PCA. **(A)** PCA of the VST transformed counts from samples across both studies and all regions. Open circles indicate samples which come from the 16 h study, closed circles indicate samples from the 24 h study. Xs mark samples which were flagged as outliers due to being more than 2 standard deviations away from the respective cluster of their region in either PC1 or PC2. **(B)** PCA performed only using the samples from each study and region, and excluding outliers from **(A)**. Abbreviations: h, hour; HFD, high-fat diet; HY, hypothalamus; HPF, hippocampus; SC, superior colliculus; IC, inferior colliculus; PC, principal component; PFC, prefrontal cortex; ZI, zona incerta.

All remaining samples were loaded into DESeq2 and pre-processed by filtering lowly expressed genes and applying VST as described in the Methods. PCA plots of the first two components revealed that samples form clusters by region and duration of study (Fig. 4A). This was used as an additional quality control metric, flagging samples as failed if they were more than two absolute standard deviations away from the mean of either PC1 or PC2 for their respective brain region (indicated by red Xs on Fig. 4A).

## Usage Notes

This research presents an extensive RNA-seq atlas of various mouse brain regions, under conditions of fasting and a HFD. This dataset can serve as a reference for researchers interested in studying the changes in gene expression programs related to energy homeostasis, specifically in the context of obesity. We have provided gene expression data from hypothalamus, hippocampus, superior colliculus, inferior colliculus, frontal cortex, and zona incerta samples of mice under varying dietary conditions.

An important consideration for researchers is the role of mouse strain and sex in the results of our study. Here, we used male C57BL6 mice and any interpretation of this data should bear this in mind as responses may vary across different strains or sexes. Additionally, we focused our attention on acute fasting and HFD exposure, which are two common manipulations in obesity research. We must underscore that our study’s diet exposure was limited to at most a 24-hour period. This short-term exposure is particularly relevant in the context of food novelty, as it could impact behaviours related to food neophobia, alter food reward processing, and influence feeding patterns, potentially leading to over or underconsumption. These aspects are relevant when considering our results, especially in relation to studies that may involve longer-term diet exposures which may act on metabolism and transcriptomics via different mechanisms. Moreover, during the 16 h study, the mice exposed to HFD consumed in average 2.7 g of HFD (5.56 kcal/gram, Research Diets D12331i), which corresponds to 15 kcal, while the mice that continued on chow diet consumed 3.5 g of chow diet (3.389 kcal/g = 11.8 kcal). Therefore, food neophobia per se was not observed, when these mice were exposed to HFD. Similarly, the amount of HFD consumed, are very comparable to the consumed amounts during chronic experiments, which excludes overconsumption for this particular experiment. Due to the high palatability of the HFD, it is likely that also neurocircuits involved in food reward has been activated. Finally, there is no overlap in sequencing between the 16 h and 24 h study, making it impossible to separate the technical effects of sequencing from those of longer exposure to fasting or single housing.

Our manuscript also offers an outline of methods used for animal handling, tissue dissection, RNA extraction, library preparation, sequencing, and data processing, which can be beneficial for researchers seeking to replicate or build upon our experimental setup. All the raw and processed data are accessible and can be used for further computational analyses, including differential gene expression analysis, network analysis, or machine learning. The data we have generated can also be used to investigate regional gene expression differences in the brain under normal chow diet conditions, as we included chow-fed controls in both of our studies.

In order to enable reuse and further exploration of the data we make available our own code and notebooks detailing how to obtain differentially expressed gene lists (Table 1**)**, gene coexpression modules using weighted correlation network analysis^22,23^ (https://github.com/perslab/wgcna-toolbox) and test whether these correlate with diet, and cell types in single-cell atlases of the mouse brain^16^.

**Table 1:** A summary of differentially expressed genes (DEGs) by study duration, region, and comparison.

Study	Region	Comparison	No. differentially expressed genes
16 h	HY	HFD.vs.chow	2
16 h	HY	fast.vs.chow	37
16 h	HPF	HFD.vs.chow	129
16 h	HPF	fast.vs.chow	6
16 h	SC	HFD.vs.chow	0
16 h	SC	fast.vs.chow	7
16 h	IC	HFD.vs.chow	0
16 h	IC	fast.vs.chow	0
16 h	PFC	HFD.vs.chow	0
16 h	PFC	fast.vs.chow	11
24 h	HY	fast.vs.chow	2728
24 h	HPF	fast.vs.chow	1019
24 h	SC	fast.vs.chow	1612
24 h	IC	fast.vs.chow	1006
24 h	ZI	fast.vs.chow	1387

Regions: hypothalamus (HY), hippocampus (HPF), superior colliculus (SC), inferior colliculus (IC), frontal cortex (PFC), or zona incerta (ZI). Comparisons of HFD or fasting versus chow diet.

## Data Availability

All code covering data pre-processing, quality control, and figures included in this manuscript are deposited and available in our Zenodo repository^20^. We include additional interactive notebooks which give users examples of how to perform differential gene expression analysis using DESeq2, discover gene modules using weighed gene co-expression analysis (WGCNA), and find enriched terms in databases.
